# Acute lung injury: how to stabilize a broken lung

**DOI:** 10.1186/s13054-018-2051-8

**Published:** 2018-05-24

**Authors:** Gary F. Nieman, Penny Andrews, Joshua Satalin, Kailyn Wilcox, Michaela Kollisch-Singule, Maria Madden, Hani Aiash, Sarah J. Blair, Louis A. Gatto, Nader M. Habashi

**Affiliations:** 1 Department of Surgery, SUNY Upstate Medical University, 750 E. Adams Street, Syracuse, NY 13210 USA; 2 Department of Biological Sciences, SUNY Cortland, Cortland, NY USA; 3 Department of Trauma Critical Care Medicine, R Adams Cowley Shock Trauma Center, University of Maryland School of Medicine, Baltimore, MD USA

**Keywords:** Acute lung injury, Injurious mechanical ventilation, TCAV protocol

## Abstract

The pathophysiology of acute respiratory distress syndrome (ARDS) results in heterogeneous lung collapse, edema-flooded airways and unstable alveoli. These pathologic alterations in alveolar mechanics (i.e. dynamic change in alveolar size and shape with each breath) predispose the lung to secondary ventilator-induced lung injury (VILI). It is our viewpoint that the acutely injured lung can be recruited and stabilized with a mechanical breath until it heals, much like casting a broken bone until it mends. If the lung can be “casted” with a mechanical breath, VILI could be prevented and ARDS incidence significantly reduced.

A patient comes into the emergency department after falling out of a tree and an x-ray confirms radial and ulnar fractures. The orthopedic physician reduces the fractured bones and places the arm in a cast. A subsequent x-ray demonstrates the fractured bones are in proper alignment with anatomic reduction. Despite the anatomic reduction, a cast is necessary to stabilize the fracture, as it will remain unstable until the bones heal and regain independent stability. Only after the healing takes place (weeks) will the cast be removed; otherwise the bone will re-fracture, exacerbating the original injury and causing further tissue injury.

This analogy illustrates the possibility that acute respiratory distress syndrome (ARDS), resulting in repetitive alveolar collapse and expansion (RACE) [1], would result in progressive tissue damage known as ventilator-induced lung injury (VILI), unless a mechanical ventilation “cast” could be applied to stabilize these alveoli, much like preventing tissue damage from an unstable broken bone. Of course maintaining an open and stable lung using mechanical ventilation is a much more difficult problem then putting a cast on a broken arm. Alveoli are inherently unstable but a delicate interplay of pulmonary surfactant function combined with mechanical support of interconnected alveolar microanatomy and non-diffusible nitrogen, result in structural interdependence and maintain an open and stable lung at a normal functional residual capacity (FRC) [2].

However, the closing capacity of the lung is altered during ARDS secondary to loss of these lung stabilizers. Pulmonary edema [3] and ventilation (spontaneous and mechanical) [4] can deactivate pulmonary surfactant function. Once developed, edema and surfactant dysfunction require time to resolve and alveolar stability to be reestablished. The existence of edema, surfactant dysfunction, and RACE cannot be identified by blood gases [5]. During ARDS, the lung is pressure dependent (i.e. will collapse at atmospheric pressure) and both time to recover and an environment conducive to regaining inherit lung stability are required before the pressure stabilizing (casting) the lung can be withdrawn.

This is exemplified in clinical trials testing the efficacy of open-lung ventilation strategies where normalized oxygenation is equated with alveolar stability, triggering a reduction in airway pressure or ventilation mode change, which occurred in the high frequency oscillatory ventilation (HFOV) trials or when using the positive end-expiratry pressure (PEEP)/fraction of inspired oxygen (FiO_2_) scale of low tidal volume (Vt) strategy [6, 7]. It is well-understood that adequate time must be given for the broken bone to heal before removing the cast; however, this same obvious concept is often overlooked when managing the “broken lung”. All too often following recruitment of the acutely injured lung there is a compulsion to reduce airway pressure as soon as oxygenation increases and the “casted” lung becomes “unstable” again with VILI-induced tissue damage recurring.

The purpose of this viewpoint paper is to consider the concept of casting a broken lung. Acute lung injury causes the loss of pulmonary stabilizers rendering the lung unstable, much like a broken bone. We hypothesize that it is possible to apply an adaptive mechanical breath in patients with ARDS that would stabilize the lung until it heals and avert a secondary VILI. Better yet, could we stabilize the lung before it even “breaks”, with a protective mechanical breath as soon as the patient is intubated and prevent early lung instability and the development of ARDS altogether?

## True lung rest and the need to establish and maintain stability

It has been theorized that ARDS causes heterogeneous lung injury with both stress-risers (i.e. collapsed or edema-filled alveoli directly adjacent to patent alveoli) and alveolar instability [8] secondary to surfactant deactivation [4]. This heterogeneous injury causes excessive alveolar strain during tidal ventilation resulting in a secondary VILI, which may lead to placing the patient with ARDS on extracorporeal membrane oxygenation (ECMO). Once on ECMO, the clinician may decide to initiate “lung rest”, a concept [9, 10] somewhat analogous to putting a cast on the lung until it heals. The problem, however, with this approach is that significant lung pathology can occur due to lung collapse alone [4, 11, 12] and the lung will eventually have to be reopened so the patient can be weaned off ECMO. Thus, resting the acutely injured lung in a collapsed state would be like casting a fractured arm without properly aligning the bones first. In addition, chronic collapse may lead to irreversible collapse induration, a form of serve alveolar fibrosis [13–15]. The lung is designed to function optimally only when fully inflated and thus the ideal situation would be to cast the open lung and let it heal at its biologically natural volume. If the open lung could be “rested” using a time-controlled adaptive ventilation (TCAV) protocol we would have the best of both worlds; VILI would be eliminated and all the negative ramifications of ECMO would be avoided.

## ARDS pathophysiology

**Fig. 1:**
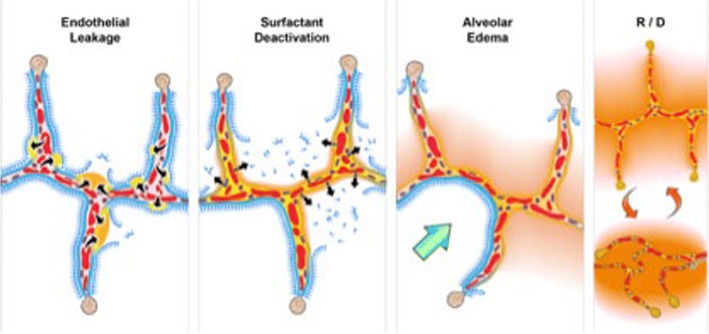
Schematic representation of the pathologic tetrad of the acute respiratory distress syndrome (ARDS). The diagram depicts multiple alveolar walls containing pulmonary capillaries (red circles), the alveolar walls are lined with a liquid hypophase (blue layer inside each alveolus), with pulmonary surfactant forming a complete monolayer on the hypophase. Severe trauma, hemorrhagic shock, or sepsis can cause the systemic inflammatory response syndrome (SIRS) that increases permeability of the pulmonary vasculature. **(Endothelial Leakage)** Increased microvascular permeability allows pulmonary edema to move into the alveolus, initially as individual blebs (increased permeability - arrows and edema blebs in tan color) [70]. **(Surfactant Deactivation)** Pulmonary surfactant molecules remain in a continuous layer initially as the edema blebs form but as the blebs expand the monolayer is disrupted leading to surfactant deactivation. **(Alveolar Edema)** A combination of the edema usurping surfactant from the hypophase, the proteins in the edema fluid deactivating surfactant [71], and improper mechanical ventilation [4] causing further surfactant disruption, leads to the destruction of the surfactant monolayer (**Surfactant Deactivation**). Loss of this monolayer results in increased alveolar surface tension causing the alveoli to become unstable and collapse at expiration (**Recruitment**/**Derecruitment** (**R**/**D**)). In addition high surface tension has been shown to increase edema flooding of the alveoli setting up a viscous cycle of edema→surfactant deactivation→high alveolar surface tension→more edema [72]. If this viscous cycle is not blocked eventually the alveolar edema will flood the entire alveolus (tan color) preventing gas exchange, leading to hypoxemia and CO_2_ retention. A hallmark of ARDS pathophysiology is heterogeneous injury with edema-filled (tan color) adjacent to air-filled alveoli with normal surfactant function (**Alveolar Edema**). Edema adjacent to air-filled alveoli create a stress-riser causing the alveolar wall to bend toward the fluid filled alveolus, which can cause stress-failure at the alveolar wall [32]. (**Green Arrow-Alveolar Edema**) Stress-risers are a key mechanism of ventilator-induced lung injury (VILI) [30–33]. Loss of surfactant function renders the alveoli unstable such that they recruit and derecruit (R/D) with each breath. The alveoli in the top frame of R/D are fully inflated but collapse during expiration in the bottom R/D frame. Alveolar R/D is another key mechanism of VILI and is known as atelectrauma [38]

There is a tetrad of pathology associated with ARDS: (1) increased pulmonary capillary permeability; (2) surfactant deactivation; (3) alveolar flooding with edema; and (4) altered alveolar mechanics with a dynamic change in alveolar size and shape with each breath (Fig. 1) [16]. The combined impact of this pathology is a significant loss of functional residual capacity (FRC) with heterogeneous lung collapse and instability with closing volume greater than FRC-producing tidal opening and closing of airspaces [17]. Although the pathophysiology of VILI is complex [18–21], in part due to the complexity of alveolar microanatomy, we postulate that ventilating the unstable lung at low lung volume is the core mechanism of VILI-induced tissue damage. In addition, raising tidal volume *without* stabilizing FRC would result in a greater number of lung units that were previously protected from mechanical ventilation with permissive atelectasis to now engage in VILI-inducing recruitment-derecruitment (R/D)-induced tissue damage [10].

**Fig. 2:**
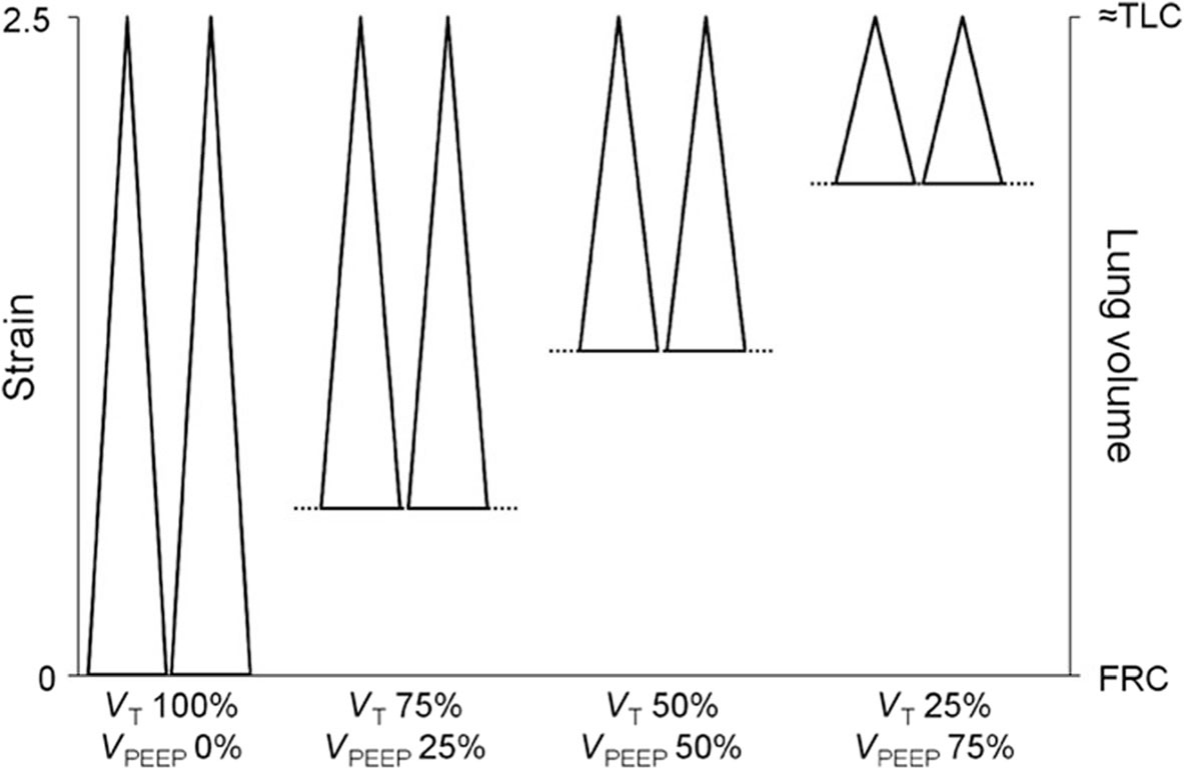
The impact of dynamic versus static lung strain on lung injury in normal pigs ventilated for 54 h. Four groups of animals were studied and in all four groups the lungs were ventilated with a very high static strain (2.5) at total lung capacity (TLC). High dynamic strain was caused by the tidal volume (V_T_) being 100% of the lung volume with no positive end-expiratory pressure (V_PEEP_). Thus there was a large change in lung volume (i.e. high dynamic strain) with each breath. In the lowest dynamic strain group V_T_ accounted for 25% of the lung volume and V_PEEP_ for 75% of the lung volume. Thus there would be a very small change in lung volume (i.e. low dynamic strain) with each breath. In the high dynamic-strain group all animals developed pulmonary edema and died before the end of the study. Conversely, none of the low dynamic-strain group developed edema and all lived until the end of the experiment [22]. This study suggest that high static strain does not damage normal lung tissue as previously hypothesize [34] but rather must be combined with a high dynamic strain to cause VILI. These data were supported in a heterogeneous porcine lung injury model (Fig. 4) in which high static strain caused no lung damage, whereas high dynamic strain injured the normal tissue and exacerbated damage in the acutely injured tissue [23]

**Fig. 3:**
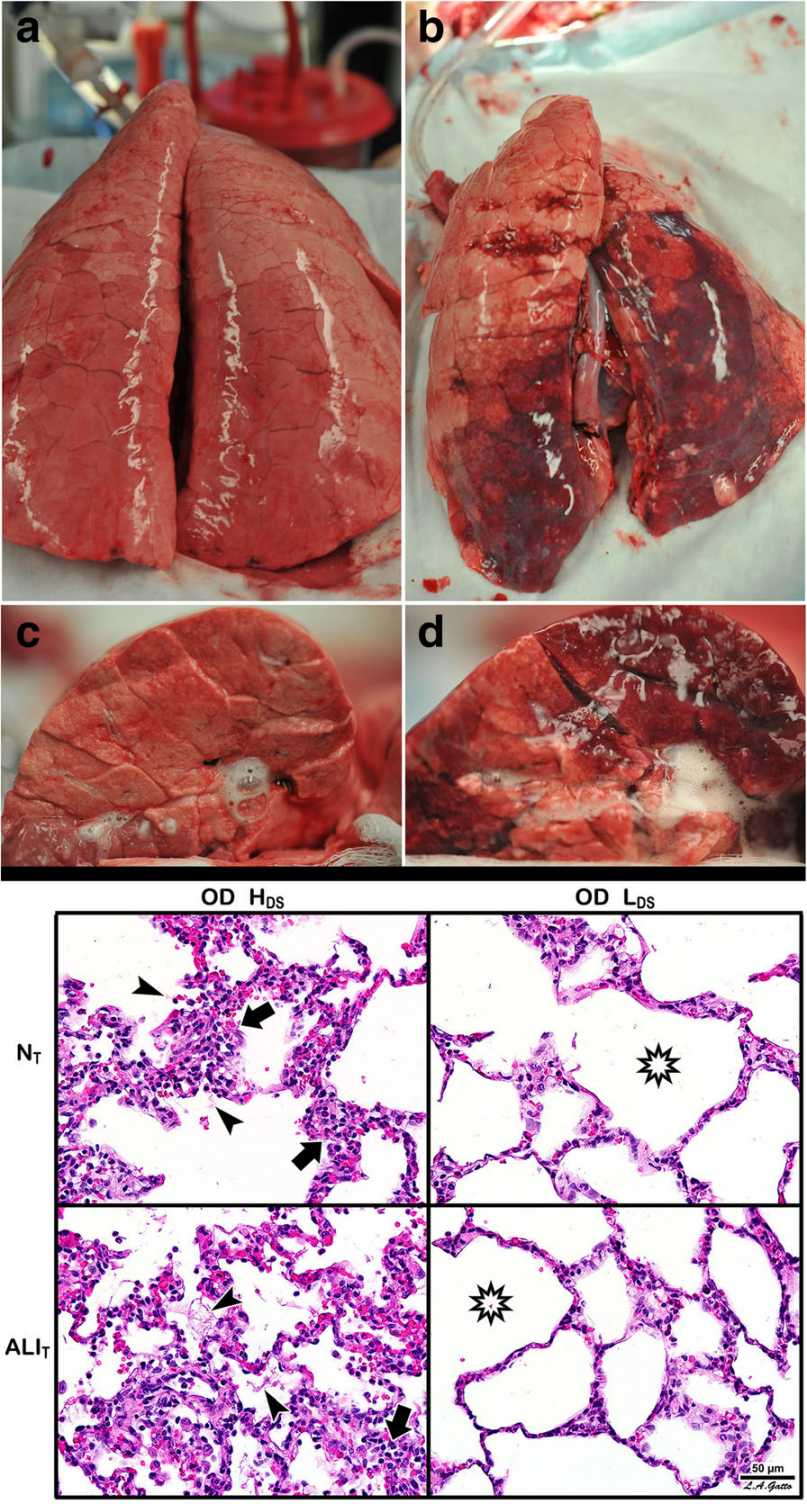
A novel heterogeneous lung injury model in which the impact of any mechanical breath can be tested in both normal (“baby lung”) and the injured (acute respiratory distress syndrome (ARDS)) lung tissues. Heterogeneous injury was caused to very specific areas of lung tissue by instillation of Tween-20 via bronchoscopy into the dependent portion of the diaphragmatic lobe with the pig in the supine position. The remaining lung tissue not exposed to Tween-20 was normal. Following Tween-20 injury animals were split into two groups: either high dynamic strain (H_DS_) caused by an extended expiratory duration or low dynamic strain (L_DS_) with a very short expiratory duration. Both groups were exposed to over-distension (plateau airway pressure 40 cmH_2_O). It is currently believed that high plateau airway pressures (≥ 30 cmH_2_O) causes ventilator-induced lung injury (VILI) in a heterogeneous ARDS lung by over-distending (OD) the remaining normal tissue (i.e. the baby lung) [34]. The goal of the study was to identify if OD would cause VILI in the baby lung, if OD would exacerbate tissue damage in the Tween-20-injured lung tissue, and if dynamic strain played a role in lung tissue injury and/or protection. **Gross Lung Photos:** The top panel (**a**-**d**) shows the whole lung and the cut lung surface at necropsy. In the OD + L_DS_ group (**a**, **c**) the lung was prevented from collapsing at expiration by using a very short expiratory duration. In the OD + H_DS_ group (**b**, **d**) the lung was allowed to collapse during expiration by extending the expiratory duration. This study demonstrates that OD did not grossly injure the normal lung tissue, nor did it exacerbate injury in the tissue injured with Tween-20 (**a**, **c**), as long as the dynamic strain was minimal. The lung is uniformly inflated (top panel **a**) and the cut lung surface appears well-inflated without interlobular edema (top panel **c**). OD combined with H_DS_ (top panel **b**, **d**) exacerbated damage in the Tween-20-injured tissue and directly injured the baby lung. The lung showed marked atelectasis, extending into the normal lobes that were not exposed to Tween 20 (top panel **b**). The cut surface showed extensive atelectasis, interlobular edema (clear jelly-like substance between lobules), and significant airway water and edema foam in the airways (top panel **d**). This study demonstrates that OD + H_DS_ exacerbated injury to the Tween-20-damaged tissue and caused direct VILI injury to the normal tissue not exposed to Tween 20, whereas OD + L_DS_ caused no injury to the baby lung and did not exacerbate injury in the Tween-20-injured tissue. **Lung Histology:** The bottom panel shows representative histology staining in both the normal tissue (N_T_) and the Tween-20-injured lung tissue (ALI_T_) in both the OD + H_DS_ and OD + L_DS_ groups. OD + H_DS_ caused severe injury to the N_T_ and exacerbated injury in the ALI_T_ tissues. Arrows indicate infiltration of inflammatory white blood cells and the arrowhead identifies the presence of fibrin deposits in the airspace (i.e pulmonary edema). This pathology was not seen in the OD + L_DS_ group and the star shows the improved alveolar patency as compared with the OD + H_DS_ group. This study suggests that OD alone does not injury the baby lung unless combined with high dynamic strain [23]

Studies have shown that elevated airway pressure with levels known to cause VILI is relatively benign if the lung is not allowed to fall significantly below FRC [22–24]. The majority of these studies used positive end-expiratory pressure (PEEP) to prevent lung collapse during expiration. Maintaining adequate FRC has also been shown to be protective in the normal lung. Pigs mechanically ventilated for 54 h at total lung capacity (TLC) with very high strain (global strain = 2.5, near TLC) do not develop ARDS as long as PEEP is sufficient to prevent lung collapse at end-expiration. Pigs with normal lungs ventilated at the same high strain (2.5) but without PEEP, allowing the lung to collapse at expiration, develop severe ARDS with a high mortality rate (Fig. 2) [22]. High ventilation driving pressure (DP) in humans, measured by dividing lung compliance (Cstat) into tidal volume (Vt) (DP = Vt/Cstat), correlates with an increase in ARDS mortality [25]. Decreasing Vt can lower DP, but that would lead to further heterogeneous lung collapse. The other solution would be to increase lung compliance, which can be accomplished by recruiting the lung. In a novel heterogeneous porcine lung injury model, Jain et al. showed that peak airway pressures of 40 cmH_2_O did not injure normal lung tissue or exacerbate damage to the acutely injured tissue as long as lung volume was maintained during expiration (Fig. 3) [23]. Thus, as long as the normal or heterogeneously injured lung is not allowed to collapse at expiration, VILI will be prevented, even with very high airway pressures and static strain.

## VILI pathophysiology

**Fig. 4:**
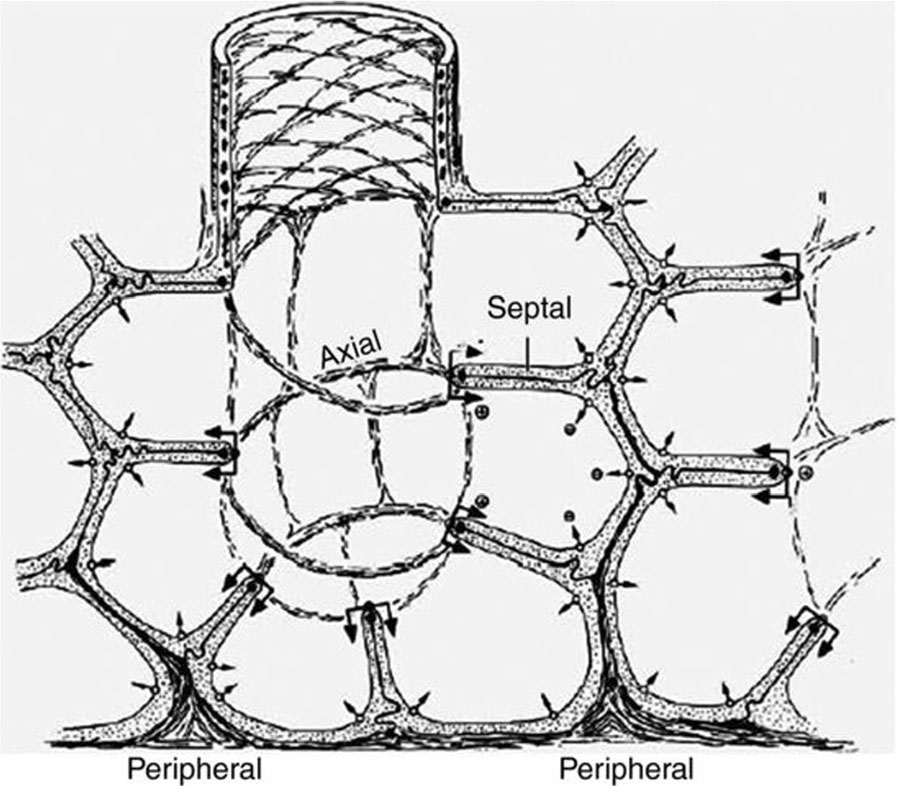
The complex interconnected structure of an alveolar sac [73]. Alveoli are not individual structures similar to a bunch of grapes but share walls with adjacent alveoli. The entire structure is bound together with a complex axial, septal, and peripheral connective tissue system. As long as all alveoli are homogenously inflated this complex structure has a great deal of stability through interdependence [26]

**Fig. 5:**
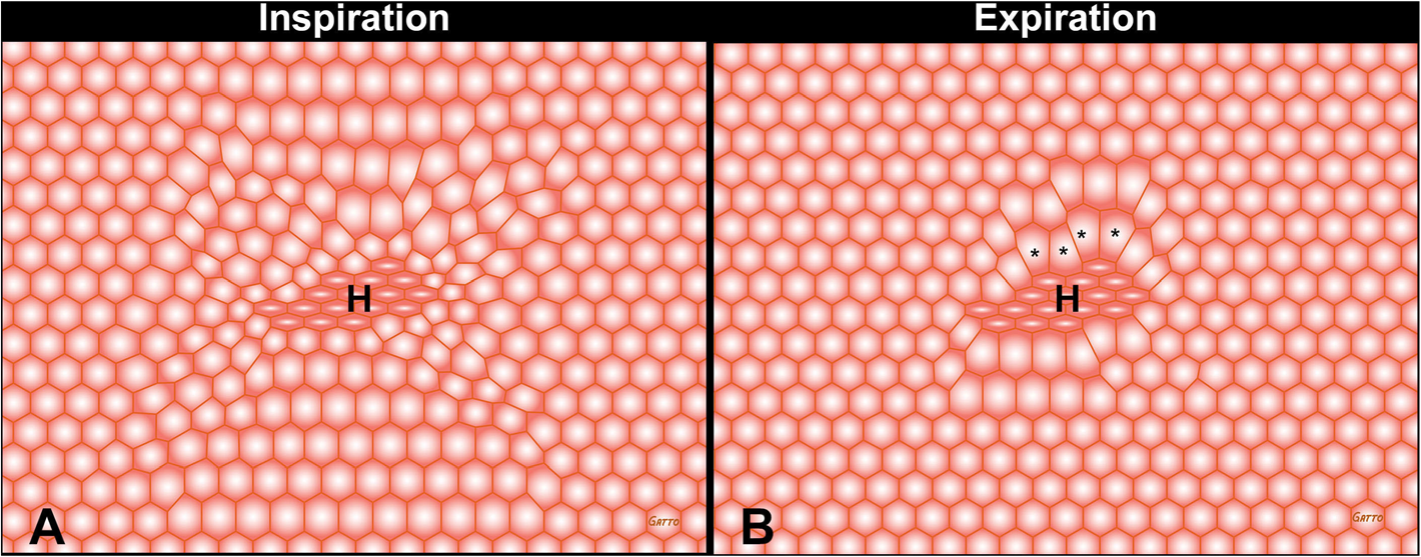
Interdependent “alveoli” with shared walls represented by hexagons at inspiration and expiration. In the center of the “alveolar tissue” there are a group of heterogeneously (H) collapsed alveoli causing a stress-riser. Since alveoli share walls, the open alveoli connected to collapsed alveoli are subject to a concentration of the force applied to lung tissue by the tidal volume. Note that the over-distension and distortion are most significant in alveoli surrounding H during expiration (asterisks). Stress-risers are a key mechanism of ventilator-induced lung injury [8]

The properly inflated lung is highly resistant to VILI due to the structural integrity of the pulmonary parenchyma known as “alveolar interdependence” (Fig. 4) [26, 27]. When alveoli and alveolar ducts collapse during expiration, this structural interdependence is lost resulting in two key pathologic changes that are primary mechanisms driving VILI: (1) lung instability with repetitive alveoli collapsing and expansion (i.e. RACE) with each breath (Fig. 1) [27–29] and (2) areas of alveoli that are adjacent to open alveoli remain collapsed throughout ventilation. The areas where collapsed and open alveoli connect are termed “stress-risers” and cause a great deal of stress in the patent alveoli (Fig. 5) [30–33]. Both of these VILI mechanisms are associated with a loss of expiratory lung volume and stability. Alveolar over-distention, the third mechanical VILI mechanism, only occurs in open alveoli adjacent to those that are collapsed (Fig. 1-Alveolar Edema and Fig. 5) [8]. This hypothesis is supported in a study showing that VILI does not occur with peak airway pressures of 40 cmH_2_O, which is believed to cause over-distension-induced lung injury, as long as the lung is fully recruited and not allowed to collapse during expiration (Fig. 3) [23]. Thus, opening and stabilizing alveoli would prevent all three mechanical mechanisms of VILI.

## We create the problem

The current standard of care is application of a protective ventilation strategy, such as low tidal volume (LVt) ventilation, *after* significant ARDS develops [34]. The progression to ARDS is often silent with normal blood gases in the presence of ARDS with unstable alveoli [35] leading clinicians to believe the lung is “fine”’ and there is no need to change to protective mechanical ventilation. Once the LVt protocol is applied, oxygenation is maintained between arterial partial pressure of oxygen (PaO_2_) of 55–80 mmHg or oxygen saturation of 88–95%, using a sliding FiO_2_/PEEP scale [34]. However, by the time this level of lung pathology is present, there is already considerable tissue and surfactant damage resulting in a significant loss of lung volume predisposing the lung to VILI [36]. Switching to the LVt protocol contributes to further loss of lung volume and PEEP, which is used in combination with the LVt protocol, may be ineffective at stabilizing alveoli as a method of lung recruitment [37, 38]. With ARDS mortality remaining unacceptably high and essentially unchanged for the past 18 years even when using the LVt protocol, new ventilation strategies are being sought [39–41].

## How to prevent the lung from “breaking”

The resolution to the problem seems to be simple; all we need to do is cast the lung at risk of developing ARDS to keep it open and stable in the event of impending lung injury. However, as ARDS progresses, vascular permeability will increase and edema fluid entering the alveolus will begin to deactivate surfactant making the lung increasingly unstable (Fig. 1). Thus the cast must be adjusted as lung pathophysiology advances or diminishes, to maintain homogeneous ventilation. Therefore, the clinician must understand how to apply the proper “dose” of the preemptive mechanical breath.

Recent work has shown that alveolar strain is viscoelastic in nature [42, 43]. The important thing to understand about a viscoelastic structure such as the alveolus, is that when force (i.e. Vt) is applied there is both a fast and slow component to alveolar opening or collapse. Thus, some alveoli might recruit in the first milliseconds (fast component) of inspiration but if the inspiratory duration is extended many more alveoli will continue to recruit (slow component). Conversely, with removal of the force (i.e. exhalation), some alveoli would begin to collapse immediately in milliseconds (fast component) but if the expiratory duration were very brief, many alveoli would simply not have time to collapse (slow component) [8].

With this knowledge, the optimal way to cast a broken lung would be with an extended time at inspiration and brief time at expiration. The extended inspiratory duration in this lung casting protocol would be a continuous positive airway pressure (CPAP). The CPAP with an open exhalation valve allows the patient to spontaneously breathe on top of the CPAP with little effort, maximizing synchrony. A very brief expiratory release time from CPAP (< 0.5 s), would not be sufficient time for the lung to completely empty.

## Time-controlled adaptive ventilation (TCAV) protocol

**Fig. 6:**
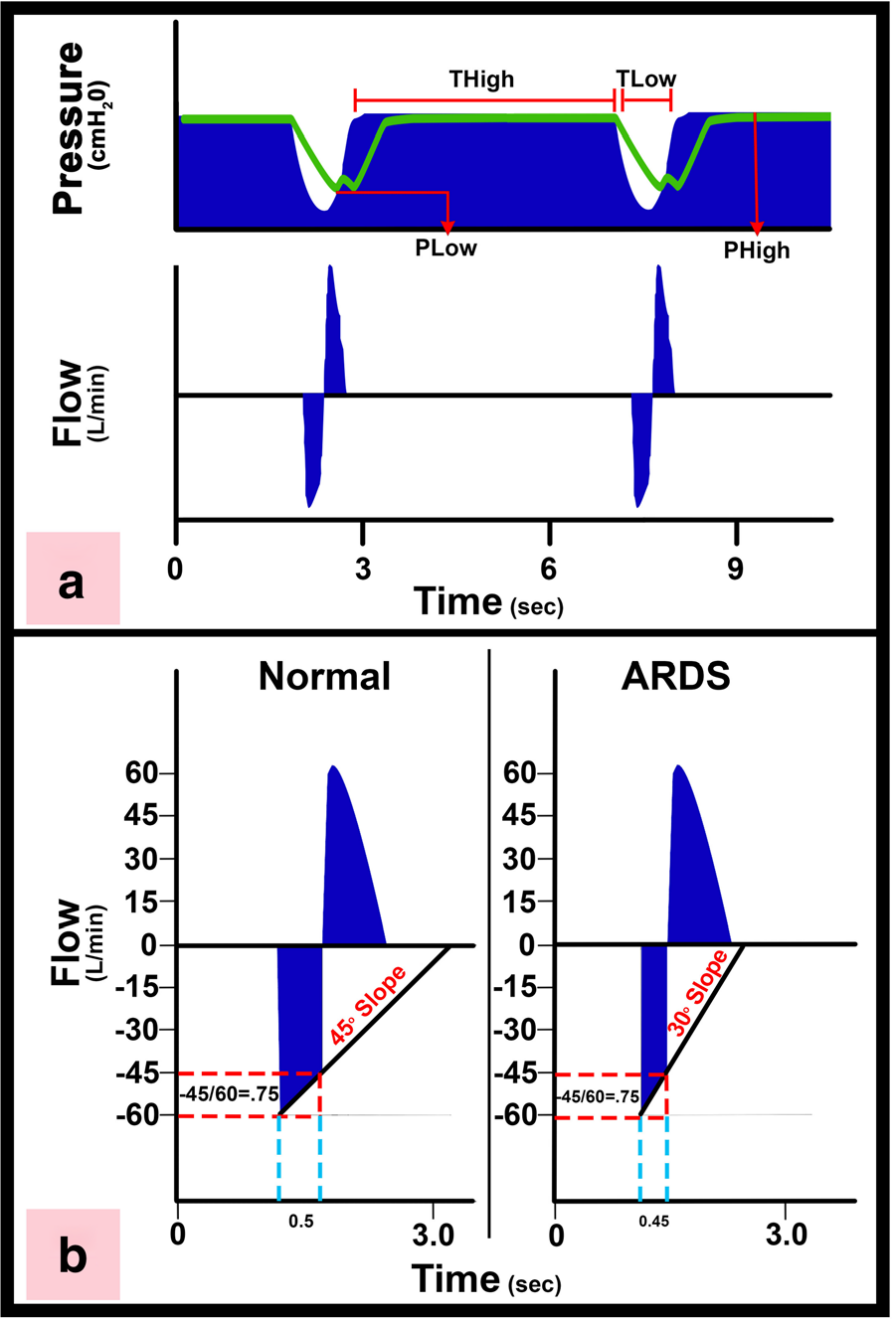
**a** Typical pressure and flow curves using the time-controlled adaptive ventilation (TCAV) protocol. There is an extended time at inspiration (T_High_) and minimal time at expiration (T_Low_). The high pressure (P_High_) combined with the T_High_ determines the magnitude and duration of the continuous positive airway pressure (CPAP). The end-expiratory airway pressure (T_Low_) is always set to 0 cmH_2_O, which minimizes the resistance to expiratory flow allowing a more accurate assessment of lung respiratory system elastance determined by the expiratory flow curve. However, P_Low_ never reaches 0 cmH_2_O because T_Low_ is set sufficiently short to maintain both lung volume and pressure at end expiration. The green line is the measured tracheal pressure, which is the actual end-expiratory pressure seen by the alveolus. We have found that if expiratory duration is set properly that the end-expiratory pressure (the actual P_Low_) is approximately ½ of the P_High_. **b** Using the slope of the expiratory flow curve (SEFC) to set the expiratory duration necessary to stabilize the lung. The SEFC of the normal lung is approximately 45°, which decreases to 30° in acute respiratory distress syndrome (ARDS). Expiratory duration is calculated by terminating expiration at 75% of the peak expiratory flow (− 60 L/min), which in this example would be at − 45 L/min. Note that using this same ratio in both normal and ARDS lungs the expiratory duration is shorter (0.45 vs. 0.5 s) in the ARDS lung because of the steeper SEFC [23]

We have developed a preemptive ventilation strategy to cast the lung maintaining homogeneous ventilation using an extended time at inspiration and a brief time at expiration [44]. The components of our TCAV protocol include the ventilator mode, the settings within this mode, and the changes in lung physiology used to modify these settings as the patient’s lung gets better or worse (Fig. 6a&b). The ventilator mode must be pressure-controlled and time-cycled, with the ability to precisely and independently control machine inspiratory and expiratory times such as airway pressure release ventilation (APRV), BiLevel, Bi-Vent, BiPhasic or DuoPAP. The settings used with our TCAV protocol include an extended time at inspiration (T_High_) that occupies ~ 90% of each respiratory cycle; the high pressure (P_High_) set sufficiently to recruit alveoli and regain FRC (Fig. 6a); the time at expiration (T_Low_) set to terminate at 75% of the peak expiratory flow rate (PEFR), which is typically ≤ 0.5 s; and the low pressure (P_Low_) set at 0 cmH_2_O (Fig. 6b). Although P_Low_ is set at 0 cmH_2_O, the pressure never reaches 0 cmH_2_O since the T_Low_ is set sufficiently brief to maintain PEEP (Fig. 6a).

Changes in lung physiology are used to adjust the settings based on assessment of the slope of the expiratory flow curve, which reflects the elastance of the respiratory system. Respiratory system resistance also determines the slope of the expiratory flow curve; however, by setting P_Low_ to zero we minimize the resistance for more accurate measurement of lung elastance. As ARDS progresses and respiratory system elastance increases, the expiratory flow slope decreases (Fig. 6b - normal lung 45° and ARDS lung 30°), and the T_Low_ is reduced to prevent these faster collapsing alveoli from de-recruiting because the ARDS lung has a faster collapse time constant [44, 45]. The TCAV protocol with the ventilator mode APRV is the same concept as the ARDSnet protocol, which includes the ventilator mode (volume-assist control), the settings within this mode (LVt < 6cc/kg, limiting plateau pressure (Pplat) < 30 cmH_2_O, etc.), and the FiO_2_/PEEP sliding scale used to adjust the settings.

Our TCAV protocol is best described as CPAP with a brief release. The CPAP phase has no trigger and patients can generate unassisted spontaneous breaths. The *time*-*controlled* component of our TCAV protocol is an extended time at inspiration (T_High_), which is greater than the slowest time constant, gradually “nudging” the lung open. The brief time at expiration, which is set less than the fastest alveolar collapse time constant, minimizes airway closure (Fig. 6b). The CPAP or P_High_ is adjusted to the pathologic condition of the patient’s lung, degree of FRC, and to changes in chest wall compliance [5]. The *adaptive* component of our TCAV protocol uses changes in lung mechanics to guide the clinician in setting the expiratory duration or T_Low_ sufficiently brief and precise to prevent derecruitment of alveoli, even those with the fastest collapse time constants (Fig. 6b) [44, 45].

In summary, the steeper the slope, the “sicker” the lung (greater respiratory system elastance) and the briefer the expiratory duration needed to prevent expiratory airway closure [46]. The TCAV protocol is so effective at recruiting lung tissue that hypercapnia is not usually a problem because there is ample alveolar surface area for CO_2_ exchange. Also, pulmonary vascular resistance (PVR) is lowest when lung volume is at FRC and thus PVR is not elevated even with the higher mean airway pressure generated with the TCAV protocol. Driving pressure remains low with the TCAV protocol even with relatively high Vt (10–12 cc/kg) since lung compliance remains normal in the fully inflated lung. Full lung recruitment also eliminates the stimulus for strong inspiratory efforts (i.e. lung stretch receptors, blood pH, PO_2_, PCO_2_ concentrations) eliminating any problem with dyssynchrony and negative pleural pressure causing pathologically high transpulmonary pressure.

**Fig. 7:**
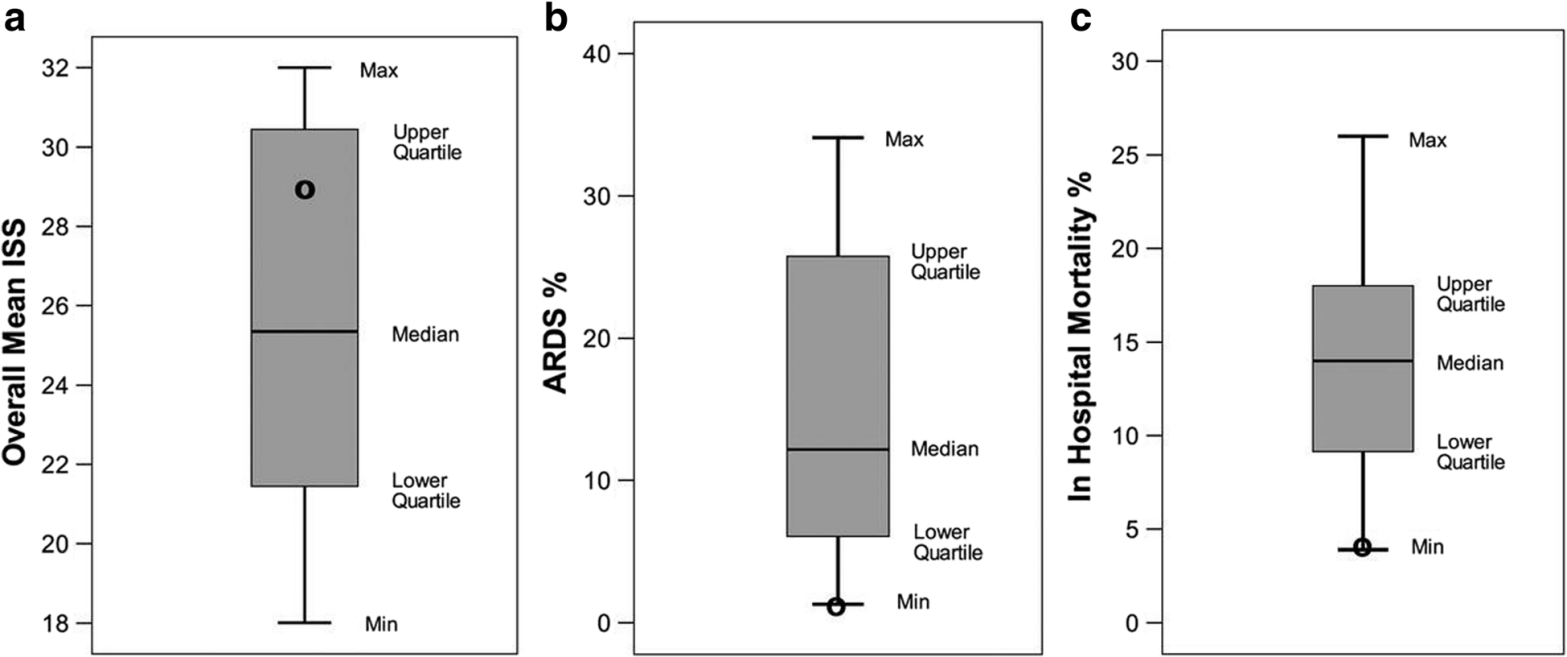
Meta-analysis comparing trauma patients in the surgical intensive care unit (SICU) in 15 university hospitals (bar and whiskers) using standard of care mechanical ventilation with patients that were placed on the time-controlled adaptive ventilation (TCAV) protocol immediately upon intubation (black circle). The injury severity score (ISS), **a**, shows that patients in the TCAV protocol (black circle) were in the upper quartile demonstrating that the positive effect was not due to the inclusion of less injured patients. Both the percentage of patients that developed acute respiratory distress syndrome (ARDS%), **b**, and the hospital mortality (In Hospital Mortality %), **c**,  were at the bottom of minimum (Min) of the bar and whisker. This study suggests that preemptive TCAV can significantly reduce ARDS incidence and mortality [47]

**Fig. 8:**
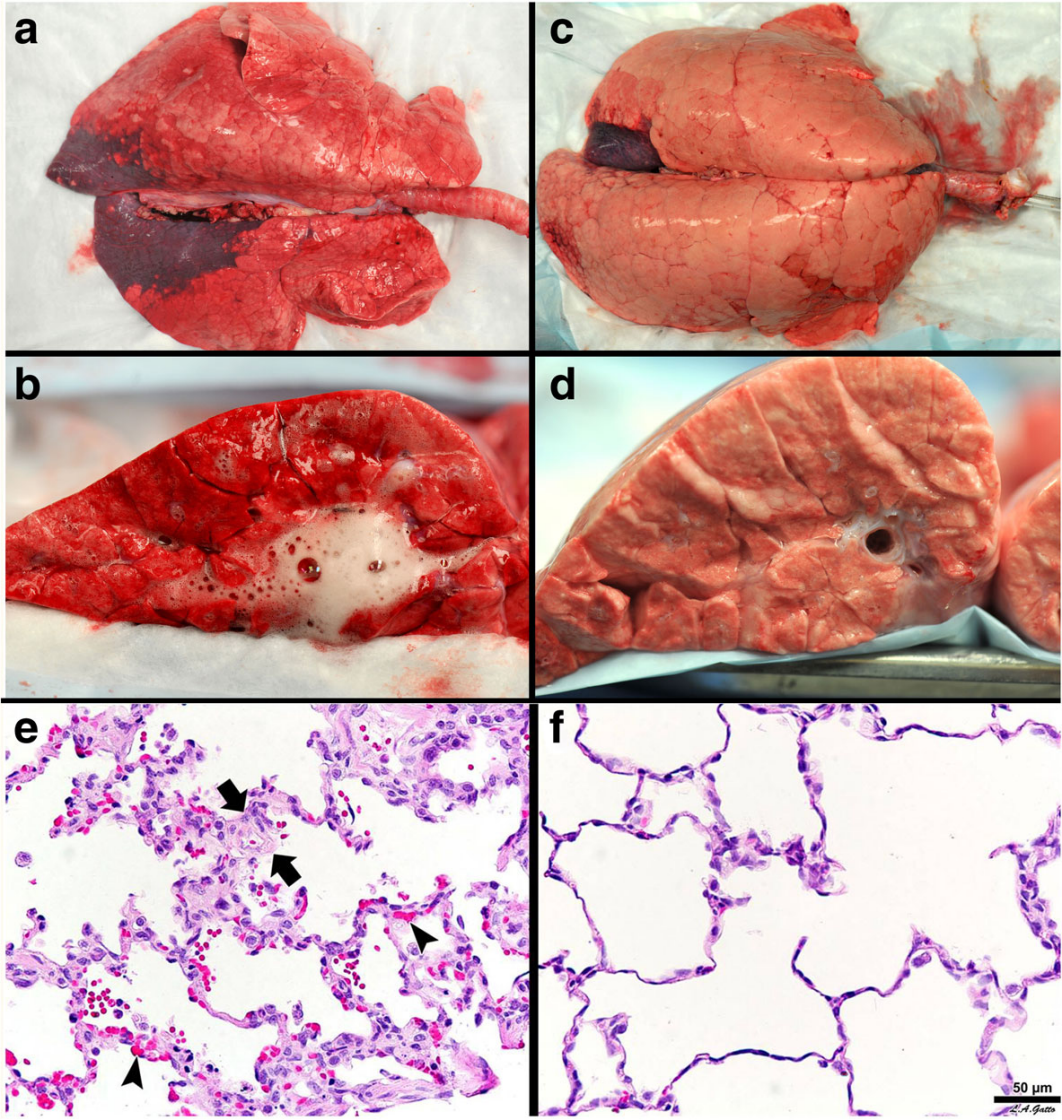
**Gross Lung Photos:** The top panel **a-d** shows gross photos of the whole lung and the lung cut surface at necropsy in a clinically applicable 48-h peritoneal sepsis plus gut ischemia/reperfusion, porcine, acute respiratory distress syndrome (ARDS) model. The lungs were inflated to 25 cmH_2_O when photographed, to standardized lung volume history (top panel **a**, **c**). One group of animals was place on the ARDSnet protocol immediately following injury (top panel **a**, **b**). The other group was placed on the time-controlled adaptive ventilation (TCAV) protocol immediately following injury (top panel **c**, **d**). Preemptive application of the ARDSnet protocol did not prevent the development of ARDS. A large area of consolidation (dark red), inflammation (reddish color), and a lung not fully inflated at an airway pressure of 25 cmH_2_O is shown (top panel **a**). The cut lung surface also demonstrated inflammation throughout the lung tissue and copious edema foam flowing from the large airways (top panel **b**). The preemptive TCAV protocol prevented the development of ARDS with the lung appearing pink (no inflammation) and fully inflated (top panel **c**). Inflated pink tissue was seen throughout the cut lung surface and no edema foam was seen in the airways (top panel **d**). **Lung Histology:**The bottom panel shows representative histology staining in the ARDSnet (**e**) and TCAV protocol (**f**). Lung tissue from the ARDSnet protocol group showed alveolar wall thickness (between arrows) and vessel congestion (arrowheads) (bottom panel **e**), which were not seen in the TCAV protocol group (bottom panel **f**) [5]

**Fig. 9:**
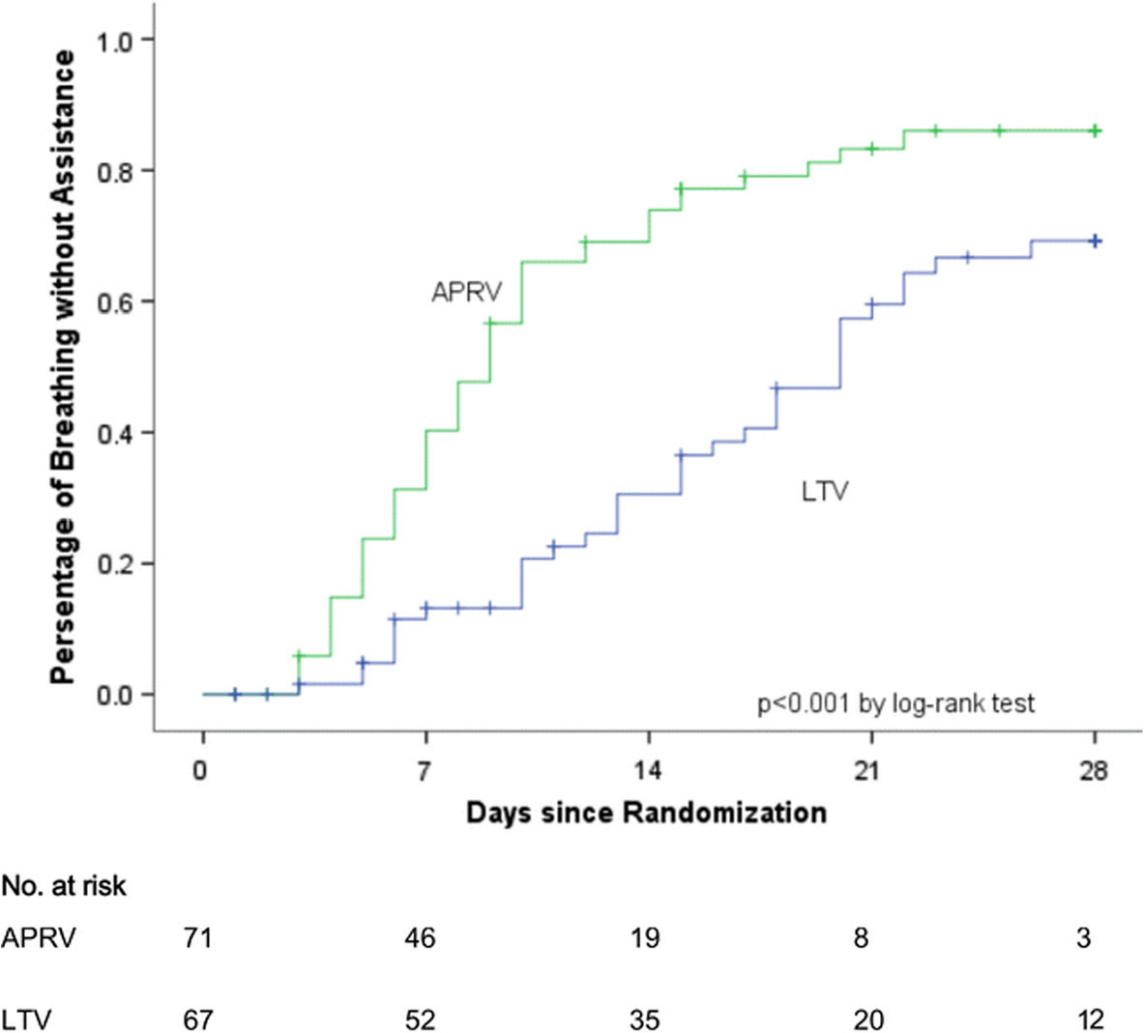
In a randomized controlled trial (RCT), patients with acute respiratory distress syndrome (ARDS) in the airway pressure release ventilation (APRV) group using a protocol similar to time-controlled adaptive ventilation (TCAV) had a reduced duration of mechanical ventilation as compared with the low tidal-volume (LTV) ARDSnet protocol group [51]

Our group has used the preemptive TCAV protocol to successfully prevent the development of ARDS in patients [47] (Fig. 7) and in translational, clinically applicable, animal models (Fig. 8) [5, 48–50]. A recent RCT using a protocol similar to TCAV reduced the duration of ICU stay and mechanical ventilation [51] (Fig. 9). Clinical settings for the TCAV protocol have been discussed in detail elsewhere [44].

## The TCAV protocol for established ARDS

Our viewpoint is that the TCAV protocol should be applied to all patients at risk of developing ARDS as soon as they are intubated. However, the TCAV protocol also works very well to open and stabilize the lungs of patients with established ARDS. Although there is not yet a RCT comparing the TCAV protocol with the ARDSnet protocol we do have strong expert experience. The TCAV protocol is the primary mode of ventilation at R. Adam Cowley Shock/Trauma Center in Baltimore, MD, USA and thus millions of hours of expert experience have been accumulated in patients with severe ARDS, including patients on extracorporeal membrane oxygenation (ECMO).

The LVt strategy is currently the standard of care because of the positive RCT showing that low (6 cc/kg) Vt versus higher (12 cc/kg) Vt significantly reduces mortality [34]. This RCT combined with evidence from other RCTs, meta-analyses, and systematic reviews have led to evidence-based medicine (EBM) guidelines strongly recommending lower Vt and plateau pressure, with moderate confidence in the effect estimates [52]. EBM has been increasingly accepted as the gold standard to direct patient care, but physicians are beginning to challenge the exclusive use of EBM to guide patient care [53–59]. A paper published in *Lancet,* a journal skeptical of the validity of EMB recommendations, criticized RCTs because they focus on internal validity (effective only on patients that fit the criteria for the controlled study) and disregard the critical issues of external validity (effective on all patients) [60]. The RCT is considered the key component of the new EBM paradigm [61]. However, EBM failures can often be attributed to this emphasis on internal validity of RCTs and thus the recommendations often fail in clinical practice (external validity) [62, 63]. Fernandez et al. stated that, “The main problem with the EMB approach is the restricted and simplistic approach to scientific knowledge, which prioritizes internal validity as the major quality of the studies to be included in clinical guidelines”. Thus, EBM suggested treatment strategies may or may not be the optimal and externally validated expert experience of physicians that have used treatment strategies successfully in their ICUs should also be considered.

The recently published ART Trial RCT applied the open lung approach (OLA) using conventional ventilation strategies (low Vt, PEEP, and recruitment maneuvers (RM)) in patients with established ARDS. In this study the OLA group had an increase in mortality, suggesting the possibility that OLA may not be an effective ventilation strategy for established ARDS [64]. However, it is not known if the OLA strategy used in this study actually recruited the lung (i.e. no lung scans showing full recruitment). We have shown that the TCAV protocol is superior to controlled mechanical ventilation (CMV) with high PEEP at opening and stabilizing subpleural alveoli [65–68]. We postulate, and our animal data [48, 50, 65–67] and expert clinical experience supports, that the TCAV protocol is far superior to CMV with RM plus PEEP at opening the non-compliant lung in ARDS. Using this modified TCAV protocol on brain-dead patients with collapsed lungs we have increased the number of transplantable lungs by ~ 680% (unpublished observations). Further support comes from a RCT comparing a protocol similar, but not identical to, TCAV with the ARDSnet protocol in patients with ARDS [51]. This studied showed that the TCAV protocol improved oxygenation and respiratory system compliance (suggesting superior lung recruitment), decreased plateau pressure and decreased both the ICU stay and duration of mechanical duration (Fig. 9). We postulate the reason why the TCAV protocol is superior at opening the lung in ARDS is due to the viscoelastic nature of alveolar opening and collapse [8]. The extended time at inspiration will recruit lung tissue at a much lower airway pressure than is needed to open the lung with a RM, since it is not just the pressure but the time the pressure is applied that recruits lung tissue [69]. Unlike a RM that is a one-time application of high airway pressure, the TCAV protocol maintains an elevated airway pressure almost continually (except for the very brief release phase) such that alveoli are gradually “nudged” open over time.

## Conclusions

Using our understanding of ARDS pathophysiology, mechanisms of VILI at the alveolar level and dynamic alveolar inflation and deflation, we postulate it is possible to stabilize the lung in patients at high risk of developing ARDS, similar to “casting” a broken bone for stability. The TCAV protocol uses a simple strategy of open-valve CPAP with a brief, intermittent release guided by changes in lung mechanics. Because the TCAV protocol can be applied as soon as intubation, very early in ARDS pathogenesis, it will effectively “Never give the lung a chance to collapse” [8] and by doing so eliminate most VILI pathophysiology. The TCAV protocol has been shown to prevent ARDS in a group of trauma patients at high risk of ARDS when applied early and used as the primary mode of mechanical ventilation at the *R Adam Cowley Shock/Trauma* in Baltimore, MA [47, 51]; a protocol similar to TCAV was shown to improve respiratory system compliance and oxygenation and reduce the duration of mechanical ventilation and the ICU stay [51].

## Abbreviations

APRV: Airway pressure release ventilation
ARDS: Acute respiratory distress syndrome
CMV: Controlled mechanical ventilation
CPAP: Continuous positive airway pressure
DP: Driving pressure
EBM: Evidence-based medicine
ECMO: Extracorporeal membrane oxygenation
EEF: End-expiratory flow
FRC: Functional residual capacity
LVt: Low tidal volume
OLA: Open lung approach
PaO_2_: Arterial partial pressure of oxygen
PEEP: Positive end-expiratory pressure
PEF: Peak expiratory flow
P_High_: Pressure at inspiration
P_Low_: Pressure at expiration
RACE: Repetitive alveolar collapse and expansion
R/D: Recruitment Derecruitment
RM: Recruitment maneuvers
SEFC: Slope of Expiratory Flow Curve
TCAV: Time-controlled adaptive ventilation
TC-PEEP: Time controlled-positive end-expiratory pressure
T_High_: Time at high pressure
TLC: Total lung capacity
T_Low_: Time at low pressure
VILI: Ventilator-induced lung injury
Vt: Tidal volume

## References

[1] Schiller HJ, McCann UG, Carney DE, Gatto LA, Steinberg JM, Nieman GF (2001). Altered alveolar mechanics in the acutely injured lung. Crit Care Med.

[2] Comroe JH (1974). Physiology of respiration; an introductory text.

[3] Hasan D, Blankman P, Nieman GF (2017). Purinergic signalling links mechanical breath profile and alveolar mechanics with the pro-inflammatory innate immune response causing ventilation-induced lung injury. Purinergic Signal.

[4] Albert RK (2012). The role of ventilation-induced surfactant dysfunction and atelectasis in causing acute respiratory distress syndrome. Am J Respir Crit Care Med.

[5] Kollisch-Singule M, Emr B, Jain SV, Andrews P, Satalin J, Liu J, Porcellio E, Kenyon V, Wang G, Marx W (2015). The effects of airway pressure release ventilation on respiratory mechanics in extrapulmonary lung injury. Intensive Care Med Exp.

[6] Young D, Lamb SE, Shah S, MacKenzie I, Tunnicliffe W, Lall R, Rowan K, Cuthbertson BH, Group OS (2013). High-frequency oscillation for acute respiratory distress syndrome. N Engl J Med.

[7] Ferguson ND, Cook DJ, Guyatt GH, Mehta S, Hand L, Austin P, Zhou Q, Matte A, Walter SD, Lamontagne F (2013). High-frequency oscillation in early acute respiratory distress syndrome. N Engl J Med.

[8] Nieman GF, Satalin J, Kollisch-Singule M, Andrews P, Aiash H, Habashi NM, Gatto LA (2017). Physiology in medicine: understanding dynamic alveolar physiology to minimize ventilator-induced lung injury. J Appl Physiol (1985).

[9] Alapati D, Aghai ZH, Hossain MJ, Dirnberger DR, Ogino MT, Shaffer TH, Extracorporeal Life Support Organization Member Centers (2017). Lung rest during extracorporeal membrane oxygenation for neonatal respiratory failure-practice variations and outcomes. Pediatr Crit Care Med.

[10] Gattinoni L, Carlesso E, Langer T (2012). Towards ultraprotective mechanical ventilation. Curr Opin Anaesthesiol.

[11] Duggan M, McCaul CL, McNamara PJ, Engelberts D, Ackerley C, Kavanagh BP (2003). Atelectasis causes vascular leak and lethal right ventricular failure in uninjured rat lungs. Am J Respir Crit Care Med.

[12] van Kaam AH, Lachmann RA, Herting E, De Jaegere A, van Iwaarden F, Noorduyn LA, Kok JH, Haitsma JJ, Lachmann B (2004). Reducing atelectasis attenuates bacterial growth and translocation in experimental pneumonia. Am J Respir Crit Care Med.

[13] Cabrera-Benitez NE, Laffey JG, Parotto M, Spieth PM, Villar J, Zhang H, Slutsky AS (2014). Mechanical ventilation-associated lung fibrosis in acute respiratory distress syndrome: a significant contributor to poor outcome. Anesthesiology.

[14] Burkhardt A (1989). Alveolitis and collapse in the pathogenesis of pulmonary fibrosis. Am Rev Respir Dis.

[15] Lutz D, Gazdhar A, Lopez-Rodriguez E, Ruppert C, Mahavadi P, Gunther A, Klepetko W, Bates JH, Smith B, Geiser T (2015). Alveolar derecruitment and collapse induration as crucial mechanisms in lung injury and fibrosis. Am J Respir Cell Mol Biol.

[16] Nieman GF, Gatto LA, Habashi NM (2015). Impact of mechanical ventilation on the pathophysiology of progressive acute lung injury. J Appl Physiol.

[17] Lambermont B, Ghuysen A, Janssen N, Morimont P, Hartstein G, Gerard P, D'Orio V (2008). Comparison of functional residual capacity and static compliance of the respiratory system during a positive end-expiratory pressure (PEEP) ramp procedure in an experimental model of acute respiratory distress syndrome. Crit Care.

[18] Slutsky AS, Ranieri VM (2013). Ventilator-induced lung injury. N Engl J Med.

[19] Uhlig U, Uhlig S (2011). Ventilator-induced lung injury. Comprehensive Physiology.

[20] Wang T, Gross C, Desai AA, Zemskov E, Wu X, Garcia AN, Jacobson JR, Yuan JX, Garcia JG, Black SM (2017). Endothelial cell signaling and ventilator-induced lung injury: molecular mechanisms, genomic analyses, and therapeutic targets. Am J Physiol Lung Cell Mol Physiol.

[21] Kuipers MT, van der Poll T, Schultz MJ, Wieland CW (2011). Bench-to-bedside review: damage-associated molecular patterns in the onset of ventilator-induced lung injury. Crit Care.

[22] Protti A, Andreis DT, Monti M, Santini A, Sparacino CC, Langer T, Votta E, Gatti S, Lombardi L, Leopardi O (2013). Lung stress and strain during mechanical ventilation: any difference between statics and dynamics?. Crit Care Med.

[23] Jain SV, Kollisch-Singule M, Satalin J, Searles Q, Dombert L, Abdel-Razek O, Yepuri N, Leonard A, Gruessner A, Andrews P (2017). The role of high airway pressure and dynamic strain on ventilator-induced lung injury in a heterogeneous acute lung injury model. Intensive Care Med Exp.

[24] Seah AS, Grant KA, Aliyeva M, Allen GB, Bates JHT (2011). Quantifying the roles of tidal volume and PEEP in the pathogenesis of ventilator-induced lung injury. Ann Biomed Eng.

[25] Amato MB, Meade MO, Slutsky AS, Brochard L, Costa EL, Schoenfeld DA, Stewart TE, Briel M, Talmor D, Mercat A (2015). Driving pressure and survival in the acute respiratory distress syndrome. N Engl J Med.

[26] Mead J, Takishima T, Leith D (1970). Stress distribution in lungs: a model of pulmonary elasticity. J Appl Physiol.

[27] Broche L, Perchiazzi G, Porra L, Tannoia A, Pellegrini M, Derosa S, Sindaco A, Batista Borges J, Degrugilliers L, Larsson A (2017). Dynamic mechanical interactions between neighboring airspaces determine cyclic opening and closure in injured lung. Crit Care Med.

[28] Farias LL, Faffe DS, Xisto DG, Santana MC, Lassance R, Prota LF, Amato MB, Morales MM, Zin WA, Rocco PR (2005). Positive end-expiratory pressure prevents lung mechanical stress caused by recruitment/derecruitment. J Appl Physiol (1985).

[29] Halter JM, Steinberg JM, Schiller HJ, DaSilva M, Gatto LA, Landas S, Nieman GF (2003). Positive end-expiratory pressure after a recruitment maneuver prevents both alveolar collapse and recruitment/derecruitment. Am J Respir Crit Care Med.

[30] Retamal J, Bergamini BC, Carvalho AR, Bozza FA, Borzone G, Borges JB, Larsson A, Hedenstierna G, Bugedo G, Bruhn A (2014). Non-lobar atelectasis generates inflammation and structural alveolar injury in the surrounding healthy tissue during mechanical ventilation. Crit Care.

[31] Chen ZL, Chen YZ, Hu ZY (2014). A micromechanical model for estimating alveolar wall strain in mechanically ventilated edematous lungs. J Appl Physiol (1985).

[32] Perlman CE, Lederer DJ, Bhattacharya J (2011). Micromechanics of alveolar edema. Am J Respir Cell Mol Biol.

[33] Makiyama AM, Gibson LJ, Harris RS, Venegas JG (2014). Stress concentration around an atelectatic region: a finite element model. Respir Physiol Neurobiol.

[34] ARDSnet (2000). Ventilation with lower tidal volumes as compared with traditional tidal volumes for acute lung injury and the acute respiratory distress syndrome. The Acute Respiratory Distress Syndrome Network. N Engl J Med.

[35] Andrews P, Sadowitz B, Kollisch-Singule M, Satalin J, Roy S, Snyder K, Gatto L, Nieman G, Habashi N (2015). Alveolar instability (atelectrauma) is not identified by arterial oxygenation predisposing the development of an occult ventilator-induced lung injury. Intensive Care Med Experimental.

[36] Force ADT, Ranieri VM, Rubenfeld GD, Thompson BT, Ferguson ND, Caldwell E, Fan E, Camporota L, Slutsky AS (2012). Acute respiratory distress syndrome: the Berlin Definition. JAMA.

[37] Lapinsky SE, Mehta S (2005). Bench-to-bedside review: recruitment and recruiting maneuvers. Crit Care.

[38] Cressoni M, Chiumello D, Algieri I, Brioni M, Chiurazzi C, Colombo A, Colombo A, Crimella F, Guanziroli M, Tomic I (2017). Opening pressures and atelectrauma in acute respiratory distress syndrome. Intensive Care Med.

[39] Maca J, Jor O, Holub M, Sklienka P, Bursa F, Burda M, Janout V, Sevcik P (2017). Past and Present ARDS mortality rates: a systematic review. Respir Care.

[40] Bellani G, Laffey JG, Pham T, Fan E, Brochard L, Esteban A, Gattinoni L, van Haren F, Larsson A, McAuley DF (2016). Epidemiology, patterns of care, and mortality for patients with acute respiratory distress syndrome in Intensive Care Units in 50 countries. JAMA.

[41] Phua J, Badia JR, Adhikari NK, Friedrich JO, Fowler RA, Singh JM, Scales DC, Stather DR, Li A, Jones A (2009). Has mortality from acute respiratory distress syndrome decreased over time?: A systematic review. Am J Respir Crit Care Med.

[42] Suki B, Barabasi AL, Lutchen KR (1994). Lung tissue viscoelasticity: a mathematical framework and its molecular basis. J Appl Physiol (1985).

[43] Suki B, Stamenovic D, Hubmayr R (2011). Lung parenchymal mechanics. Compr Physiol.

[44] Habashi NM (2005). Other approaches to open-lung ventilation: airway pressure release ventilation. Crit Care Med.

[45] Jain SV, Kollisch-Singule M, Sadowitz B, Dombert L, Satalin J, Andrews P, Gatto LA, Nieman GF, Habashi NM (2016). The 30-year evolution of airway pressure release ventilation (APRV). Intensive Care Med Exp.

[46] Brody AW (1954). Mechanical compliance and resistance of the lung-thorax calculated from the flow recorded during passive expiration. Am J Phys.

[47] Andrews PL, Shiber JR, Jaruga-Killeen E, Roy S, Sadowitz B, O'Toole RV, Gatto LA, Nieman GF, Scalea T, Habashi NM (2013). Early application of airway pressure release ventilation may reduce mortality in high-risk trauma patients: a systematic review of observational trauma ARDS literature. J Trauma Acute Care Surg.

[48] Roy S, Habashi N, Sadowitz B, Andrews P, Ge L, Wang GR, Roy P, Ghosh A, Kuhn M, Satalin J (2013). Early airway pressure release ventilation prevents ARDS-a novel preventive approach to lung injury. Shock.

[49] Roy S, Sadowitz B, Andrews P, Gatto LA, Marx W, Ge L, Wang GR, Lin X, Dean DA, Kuhn M (2012). Early stabilizing alveolar ventilation prevents acute respiratory distress syndrome: a novel timing-based ventilatory intervention to avert lung injury. J Trauma Acute Care Surg.

[50] Silva PL, Cruz FF, Samary CDS, Moraes L, de Magalhaes RF, Fernandes MVS, Bose R, Pelegati VB, Carvalho HF, Capelozzi VL, et al. Biological response to time-controlled adaptive ventilation depends on acute respiratory Distress syndrome etiology. Crit Care Med. 2018. 10.1097/CCM.0000000000003078 29485489

[51] Zhou Y, Jin X, Lv Y, Wang P, Yang Y, Liang G, Wang B, Kang Y (2017). Early application of airway pressure release ventilation may reduce the duration of mechanical ventilation in acute respiratory distress syndrome. Intensive Care Med.

[52] Fan E, Del Sorbo L, Goligher EC, Hodgson CL, Munshi L, Walkey AJ, Adhikari NKJ, Amato MBP, Branson R, Brower RG (2017). An Official American Thoracic Society/European Society of Intensive Care Medicine/Society of Critical Care Medicine clinical practice guideline: mechanical ventilation in adult patients with acute respiratory distress syndrome. Am J Respir Crit Care Med.

[53] De Vreese L (2011). Evidence-based medicine and progress in the medical sciences. J Eval Clin Pract.

[54] Holmes D, Murray SJ, Perron A, Rail G (2006). Deconstructing the evidence-based discourse in health sciences: truth, power and fascism. Int J Evid Based Healthc.

[55] Pope C (2003). Resisting evidence: the study of evidence-based medicine as a contemporary social movement. Health.

[56] Miles A, Polychronis A, Grey JE (2006). The evidence-based health care debate - 2006. Where are we now?. J Eval Clin Pract.

[57] Wyer PC, Silva SA (2009). Where is the wisdom? I--a conceptual history of evidence-based medicine. J Eval Clin Pract.

[58] Oliver K, Lorenc T, Innvaer S (2014). New directions in evidence-based policy research: a critical analysis of the literature. Health Res Policy Syst.

[59] Fernandez A, Sturmberg J, Lukersmith S, Madden R, Torkfar G, Colagiuri R, Salvador-Carulla L (2015). Evidence-based medicine: is it a bridge too far?. Health Res Policy Syst.

[60] Rothwell PM (2005). External validity of randomised controlled trials: “to whom do the results of this trial apply?”. Lancet.

[61] Montori VM, Guyatt GH (2008). Progress in evidence-based medicine. JAMA.

[62] Metge CJ (2011). What comes after producing the evidence? The importance of external validity to translating science to practice. Clin Ther.

[63] Steckler A, McLeroy KR (2008). The importance of external validity. Am J Public Health.

[64] Cavalcanti AB, Suzumura EA, Laranjeira LN, Paisani DM, Damiani LP, Guimaraes HP, Romano ER, Regenga MM, LNT T, Writing Group for the Alveolar Recruitment for Acute Respiratory Distress Syndrome Trial I (2017). Effect of lung recruitment and titrated positive end-Expiratory pressure (PEEP) vs low PEEP on mortality in patients with acute respiratory distress syndrome: a randomized clinical trial. JAMA.

[65] Kollisch-Singule M, Jain S, Andrews P, Smith BJ, Hamlington-Smith KL, Roy S, DiStefano D, Nuss E, Satalin J, Meng Q (2016). Effect of airway pressure release ventilation on dynamic alveolar heterogeneity. JAMA Surg.

[66] Kollisch-Singule M, Emr B, Smith B, Roy S, Jain S, Satalin J, Snyder K, Andrews P, Habashi N, Bates J (2014). Mechanical breath profile of airway pressure release ventilation: the effect on alveolar recruitment and microstrain in acute lung injury. JAMA Surg.

[67] Kollisch-Singule M, Emr B, Smith B, Ruiz C, Roy S, Meng Q, Jain S, Satalin J, Snyder K, Ghosh A (2014). Airway pressure release ventilation reduces conducting airway micro-strain in lung injury. J Am Coll Surg.

[68] Roy SK, Emr B, Sadowitz B, Gatto LA, Ghosh A, Satalin JM, Snyder KP, Ge L, Wang G, Marx W (2013). Preemptive application of airway pressure release ventilation prevents development of acute respiratory distress syndrome in a rat traumatic hemorrhagic shock model. Shock.

[69] Albert SP, DiRocco J, Allen GB, Bates JH, Lafollette R, Kubiak BD, Fischer J, Maroney S, Nieman GF (2009). The role of time and pressure on alveolar recruitment. J Appl Physiol.

[70] Parker JC, Townsley MI (2004). Evaluation of lung injury in rats and mice. Am J Physiol Lung Cell Mol Physiol.

[71] Taeusch HW, Bernardino de la Serna J, Perez-Gil J, Alonso C, Zasadzinski JA (2005). Inactivation of pulmonary surfactant due to serum-inhibited adsorption and reversal by hydrophilic polymers: experimental. Biophys J.

[72] Nieman GF, Bredenberg CE (1985). High surface tension pulmonary edema induced by detergent aerosol. J Appl Physiol.

[73] Weibel ER (1984). The pathway for oxygen: structure and function in the mammalian respiratory system.

